# The potential of memory enhancement through modulation of perineuronal nets

**DOI:** 10.1111/bph.14672

**Published:** 2019-05-20

**Authors:** James A. Duncan, Richard Foster, Jessica C.F. Kwok

**Affiliations:** ^1^ School of Chemistry University of Leeds Leeds UK; ^2^ Astbury Centre for Structural Molecular Biology University of Leeds Leeds UK; ^3^ School of Biomedical Sciences University of Leeds Leeds UK; ^4^ Institute of Experimental Medicine Czech Academy of Science Prague Czechia

## Abstract

With an increasingly aging global population, the incidence of neurological diseases such as dementia is set to increase to unmanageable levels, yet there are currently only symptomatic therapies available for treatment. The mechanisms underlying the development of some forms of dementia, such as Alzheimer's disease (AD), are not yet completely elucidated with several competing hypotheses existing. During the closure of the critical period in the brain, significant compositional changes occur to the neural extracellular matrix (ECM). Specifically, condensed mesh‐like structures called perineuronal nets (PNNs) form around subsets of neurons and have a profound effect on axonal growth and limit neuronal plasticity. These PNNs act as a morphological checkpoint and can influence memory and cognition. Manipulating these important ECM structures may provide the key to reactivating plasticity and restoring memory, both of which are severely impaired in AD and other associated neurological diseases. This review explores the current understanding of how PNNs are manipulated and examines potential new methods for PNN modulation.

**Linked Articles:**

This article is part of a themed section on Therapeutics for Dementia and Alzheimer's Disease: New Directions for Precision Medicine. To view the other articles in this section visit http://onlinelibrary.wiley.com/doi/10.1111/bph.v176.18/issuetoc

**Abbreviations**
ACAN: aggrecan
AD: Alzheimer's disease
BCAN: brevican
CS: chondroitin sulfate
CSPGs: chondroitin sulfate proteoglycans
ECM: extracellular matrix
GAG: glycosaminoglycan
GalNAc: *N*‐acetylgalactosamine
GlcA: glucuronic acid
HA: hyaluronan
Hapln: hyaluronan and proteoglycan link protein
NCAN: neurocan
PNNs: perineuronal nets
Tn‐R: tenascin‐R

## 1 INTRODUCTION

Alzheimer's disease (AD) is the most common form of dementia and is clinically characterised by a progressive loss of memory and functional cognition as well as other non‐cognitive disturbances such as anxiety and delusions (Yiannopoulou & Papageorgiou, 2013). The leading risk factor for AD is age, one in 23 people over the age of 65 suffer from this form of dementia (Prince et al., 2014). As our understanding of the disease state has expanded, four main hypotheses of the cause of AD have been developed: the cholinergic hypothesis, the amyloid hypothesis, the Tau hypothesis, and the genetic hypothesis. Since its initial discovery over a century ago, a definitive cure for AD is still to be found, despite numerous attempts at realising one. Regrettably, the few treatments currently available are not considered to be disease‐modifying in nature but rather, symptomatic and have been the first line of treatment for the last 30 years (Ballard et al., 2011; Cummings, Morstorf, & Zhong, 2014).

In the last two decades, attention has shifted towards treating the more recently appreciated disease pathologies, notably targeting and preventing the build‐up of amyloid β (Aβ) proteins (Selkoe & Hardy, 2016) and the microtubule associated phosphoprotein, Tau (Iqbal, Liu, Gong, & Grundke‐Iqbal, 2010), both of which are increasingly becoming explicitly linked to one another (Bloom, 2014). Several anti‐Aβ monoclonal antibody therapies (Doody et al., 2014; Vandenberghe et al., 2016) and a selection of β‐secretase (BACE1) inhibitors (Vassar, 2014) have progressed as far as phase III clinical trials but have failed to translate into commercial medicines. These recent high‐profile failures have led some to believe that new hypotheses of AD are required, as the current understanding has provided no progress to a medicine (Kepp, 2017). This has led researchers to inspect features of the brain other than the neural cells, such as the extracellular matrix (ECM), for novel inspiration.

Synaptic plasticity plays a key role in memory throughout the critical period of development, during intense periods of learning and as we age. Recent experiments have shown that neural plasticity can be restored to a juvenile‐like state through modulation of a neuronal ECM component known as perineuronal nets (PNNs), resulting in the restoration of cognition in a mouse model (Rowlands et al., 2018; Sorg et al., 2016; Yang et al., 2017). In this review, we discuss the emerging role that PNNs play in controlling plasticity and memory, presenting the unique structural and functional features of these complex ECM components and the evolving methodologies used to modulate them.

## 2 KEY COMPONENTS AND STRUCTURE OF PNNS

**Figure 1:**
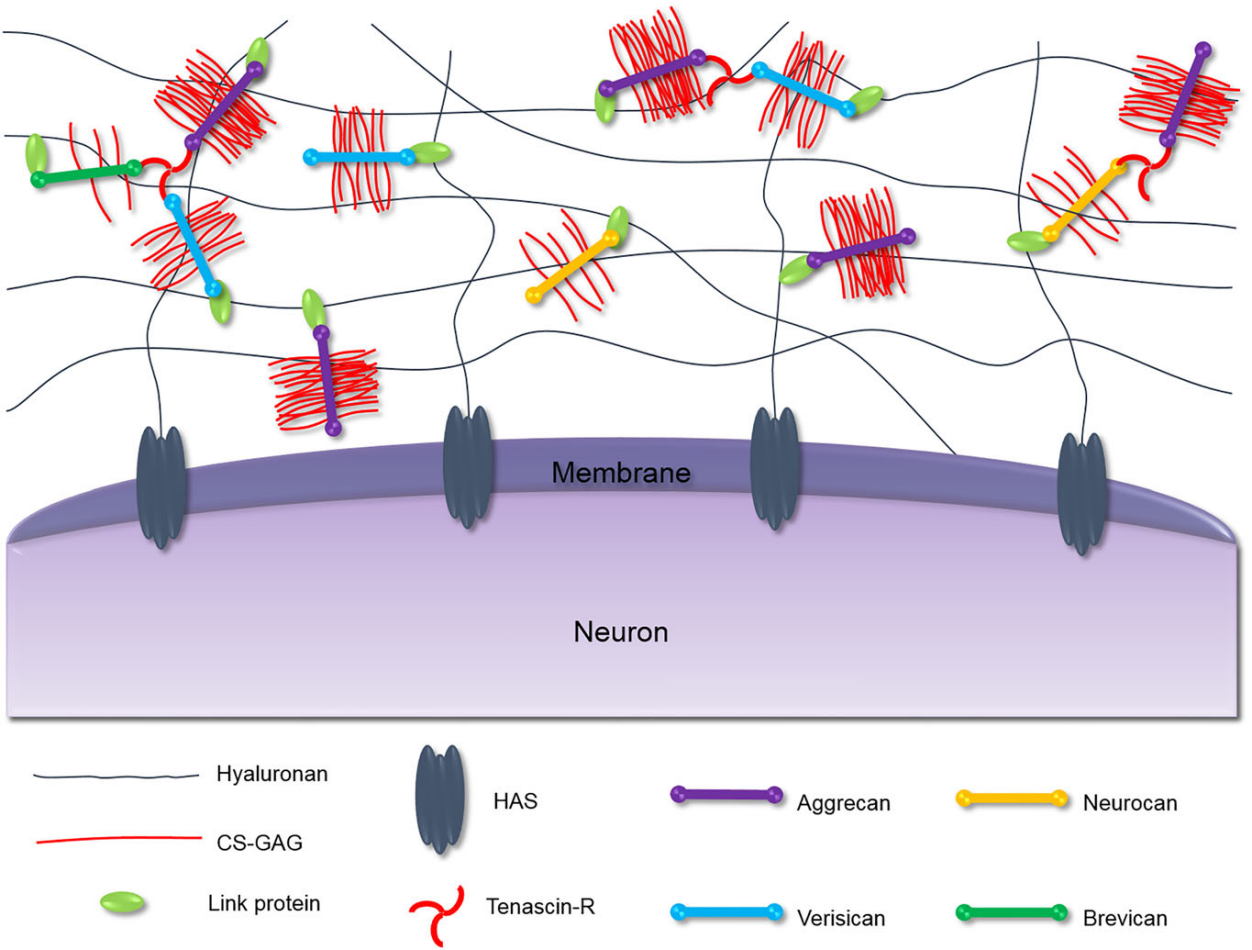
Composition of the PNNs around the neuron. The HA forms a mesh‐like backbone to which other ECM molecules can bind. HA is attached to the cell surface through HAS as well as other cell surface HA receptors (not shown). The CSPGs ‐ aggrecan, verisican, brevican and neurocan ‐ are all able to attach to chains of HA through link proteins. The CNS‐exclusive tenascin, TN‐R, can conjugate up to three lecticans, enhancing the overall rigidity of the PNNs

Neural cells produce specialised and distinctive ECM molecules that fill the diffuse space between neurons and glial cells (Gundelfinger, Frischknecht, Choquet, & Heine, 2010). These molecules can condense and ensheath specific neurons forming PNNs. These nets were first observed by Camillo Golgi in 1889, but only recently has there been an interest in their structure and function (Spreafico, De Biasi, & Vitellaro‐Zuccarello, 1999). The revelation that PNNs play a significant role in regulating plasticity and memory has intensified research efforts into elucidating their molecular composition and connectivity in recent years. PNNs are composed of several components, the structure of which is shown in Figure 1 (Kwok, Dick, Wang, & Fawcett, 2011). Hyaluronan (HA), the most abundant and most crucial component of PNNs, forms a backbone mesh‐like structure which allows for the binding of other important components such as chondroitin sulfate proteoglycans (CSPGs) and ultimately dictates the overall structure of the ECM (McRae & Porter, 2012). HA consists of alternating *N*‐acetylglucosamine and glucuronic acid (GlcA) units forming long non‐sulfated polysaccharide chains which vary in length, ranging from 25 to 1,000 kDa (Viapiano & Matthews, 2006). HA is synthesised by the transmembrane enzyme hyaluronan synthase (HAS) which anchors the PNNs to the neuronal surface (Kwok, Carulli, & Fawcett, 2010). Lecticans are the major CSPG family in the brain ECM and are able to bind an array of matrix molecules; as a result, these CSPGs are considered the organisers of the ECM (Yamaguchi, 2000). The lectican family include the non‐CNS‐specific lecticans: aggrecan (ACAN) and versican (Glumoff, Savontaus, Vehanen, & Vuorio, 1994; Popp, Andersen, Maurel, & Margolis, 2003), and the CNS‐specific lecticans: neurocan (NCAN) and brevican (BCAN; Watanabe et al., 1995; Yamada, Watanabe, Shimonaka, & Yamaguchi, 1994).

**Figure 2:**
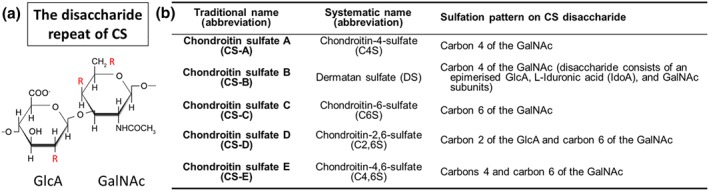
(a) The disaccharide repeat of CS. (b) Traditional and systematic nomenclature for various forms of CS present in the ECM

Lecticans are different from other CSPGs in the ECM as they are composed of globular domains enabling them to interact with HA and tenascin‐R (Tn‐R) simultaneously. All lecticans have an N‐terminal globular (G1) domain containing an immunoglobulin‐like loop repeat capable of binding HA chains and certain link proteins (Haplns) and up to two link modules at the C‐terminus (Yamaguchi, 2000). The central protein core has covalently attached chondroitin sulfate glycosaminoglycan (CS‐GAG) chains which extend in a brush‐like manner perpendicular to the core protein. The CS‐GAG chains are composed of alternating *N*‐acetylgalactosamine (GalNAc) and GlcA (Figure 2a). These extensions can be sulfated at various positions, typically carbon 4 and/or carbon 6 of the GalNAc subunit and/or carbon 2 of the GlcA subunit, which gives rise to multiple versions of chondroitin sulfate (CS) shown in Figure 2b (Mikami & Kitagawa, 2013; Silbert & Sugumaran, 2002). The CS chains vary in number, length, and pattern of sulfation, and this has a significant effect on their functions (Bandtlow & Zimmermann, 2000). The C‐terminal globular (G3) domain allows for the binding of tenascins—typically, this is Tn‐R in the PNNs. As a trimeric modular glycoprotein, Tn‐R serves to strengthen the overall macromolecular structure of PNNs through binding multiple lecticans (Lundell et al., 2004; Figure 1). Visualisation of the PNNs is commonly done using the lectin Wisteria floribunda agglutinin (WFA) staining; however, it is still unclear which component of the nets WFA binds to (Härtig, Brauer, & Brückner, 1992).

## 3 THE ROLE OF PNNS IN PLASTICITY AND MEMORY

### 3.1 Plasticity

Plasticity is the ability of neurons to reorganise and reassemble synaptic connectivity in response to experiences and external stimuli and is governed by a wide range of interrelated factors. It was previously thought that neurons and glial cells which constitute much of the brain volume were the exclusive directors of this adaptability. However, ECM molecules which form the vital links between these cells are increasingly becoming associated with neuroplasticity. As the critical period closes for plasticity, PNNs rapidly form predominantly around parvalbumin‐positive (PV+) GABAergic interneurons. This creates a lattice structure which blocks the formation of new synapses (Deepa et al., 2006; Kosaka & Heizmann, 1989; Tsien, 2013).

These lattice structures are dynamic, being turned over throughout the lifetime of the neuron, regulating communication, and acting as the gateway to the neuron. Several recent studies have provided insight into the multifaceted role PNNs play in controlling plasticity. The PNNs act not only as a physical barrier between the neurons and the rest of the ECM but also as a mediator in the binding and movement of critical binding proteins and membrane bound neuronal proteins, respectively (Dick et al., 2013; Frischknecht et al., 2009). The PNNs can be considered to act as a cationic buffer for the neurons, as they possess an unusually high negative charge density (Morawski et al., 2015). This provides protection from oxidative stress caused by cations such as Fe^3+^ (Härtig et al., 1999; Suttkus et al., 2014). Additionally, the repulsive axon guidance molecule semaphorin 3A (Sema3A) specifically binds to the glycosaminoglycan (GAG) chondroitin‐4,6‐sulfate (C4,6S), which is enriched in the PNNs. When bound to PNNs, Sema3A enhances the inhibition of PNNs to neuronal growth and is involved in restricting plasticity (Boggio et al., 2019; Dick et al., 2013). Moreover, PNNs limit the lateral movement of AMPA‐type glutamate receptors on the cell surface. Removing PNNs allows these receptors to diffuse laterally, leading to an increased paired‐pulse ratio, a readout of short‐term synaptic plasticity and is recorded using whole‐cell patch clamp. This suggests that suppressing membrane protein mobility is another way in which PNNs inhibit synaptic plasticity (Frischknecht et al., 2009).

The important role PNNs play in regulating plasticity has been shown using several animal models with focus on the visual cortex. The first evidence of this was seen in dark‐rearing experiments using cats and mice. The visual cortex critical period was extended through enhanced plasticity as a result of attenuating the expression of CSPGs and stalling PNN formation (Lander, Kind, Maleski, & Hockfield, 1997; Pizzorusso et al., 2002). Additionally, PNN formation is hindered by reducing overall neuronal activity and thus providing a potential continuation of the critical period. Using organotypic mouse brain slices and non‐specific suppression of neuronal activity by blocking voltage‐gated sodium channels, Reimers, Hartlage‐Rübsamen, Brückner, and Roßner (2007) were able to postpone the development of PNNs and maintain synaptic plasticity. Moreover, through a knockout (KO) mouse model, the *hapln1* gene which encodes the vital PNN component hyaluronan and proteoglycan link protein 1 (Hapln1), the development of PNNs can be attenuated, leading to the adult mice visual and somatosensory systems plasticity being greatly enhanced to levels that are comparable to juvenile animals (Carulli et al., 2010). Finally, adult rats suffering from amblyopia, a visual acuity disorder developed during the critical period for vision, were provided with a stimuli‐enriched environment resulting in the reduction of the density of the PNNs as well as the restoration of visual acuity and ocular dominance (Sale et al., 2007). These few examples provide compelling evidence for the close relationship thought to exist between PNNs and brain plasticity.

### 3.2 Memory

Synaptic plasticity has a long history of being linked to the encoding, storage, and retrieval of information in the form of memory (Hebb, 1949; Jones, 1994; Martin, Grimwood, & Morris, 2000). As a result, PNNs have been implicated in controlling various forms of memory. Recently, it has been reported that digestion of the PNNs wrapped around neurons in the secondary visual cortex (V2L) interrupts the recall of long‐term fear memory in rats. In contrast, more recent fear memory was undisturbed by the same change to the ECM (Thompson et al., 2017). Several studies prior to this work also showed similar remote fear memory recall impairment when PNNs were disrupted in various regions of the brain (Gogolla, Caroni, Lüthi, & Herry, 2009; Hylin, Orsi, Moore, & Dash, 2013). This suggests that PNNs stabilise existing synaptic connections and block the formation of new synapses between these neurons.

PNNs in the perirhinal cortex have also been shown to affect a different form of memory known as object recognition (OR) memory in mice. Two mouse models with Tau pathology showing significant impairment in OR memory were injected with chondroitinase ABC (ChABC) at the site of the perirhinal cortex, in order to enzymically digest the PNNs present. One week after treatment, the Tau mice demonstrated similar levels of OR memory and synaptic transmission to control animals, suggesting that ChABC may be effective in restoring memory loss in neurodegenerative disorders such as AD (Yang et al., 2015). A different study sought to genetically attenuate PNNs and investigate the effects this had on long‐term OR memory. Using the same adult *hapln1* KO mouse model used by Carulli and colleagues to investigate the effects of PNN removal on plasticity, OR memory was greatly enhanced in the absence of PNNs (Romberg et al., 2013). Furthermore, both perirhinal basal synaptic transmission and long‐term depression were measured, following on from the notion that these are the core physiological mechanisms underpinning long‐term OR memory. These parameters were enhanced by the removal of PNNs (Romberg et al., 2013).

This evidence inevitably guides research towards novel methods of altering the PNNs to enhance plasticity for memory‐related deficiencies. As previously mentioned, the most telling and disruptive symptom of AD is a loss of memory. Modulation of the PNNs may provide improvements to impaired neuronal connectivity seen in AD patients' brains, altogether bypassing the pathologies of AD.

## 4 MODULATION OF THE PNNS

### 4.1 Removing PNNs

**Figure 3:**
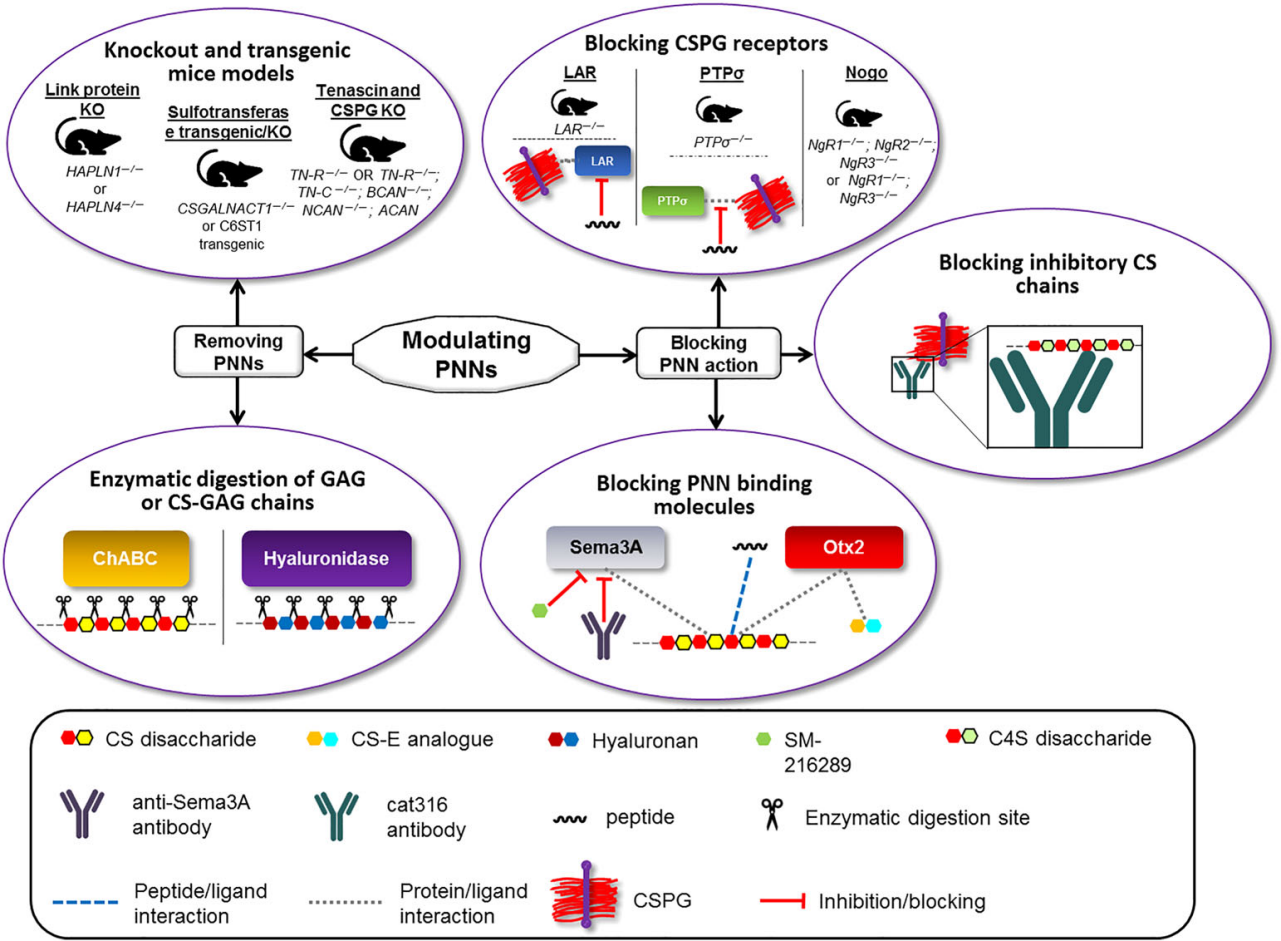
Summary of current methods for modulating PNNs

Several molecular structures including HA backbone, link proteins such as Hapln1 and Tn‐R, and the major CSPGs are essential for maintaining the structure and function of the PNNs (Kwok et al., 2010; Suttkus et al., 2014). As these components are exposed to the diffuse ECM, many extracellular macromolecules can recognise and bind to specific molecular sequences (Figure 3). An example of this is the bacterial enzyme ChABC which can indiscriminately recognise and digest CS‐GAG chains present on the CSPGs into disaccharides and partially digest HA (Saito & Yamagata, 1968). Without the CS and HA chains, the structural integrity of the CSPG is compromised resulting in a complete collapse of the PNN structure into diffuse ECM. ChABC has been used extensively in a range of experiments to investigate the effects of PNN removal on various parameters and indications including plasticity, memory, and spinal cord injury recovery (Bradbury et al., 2002; Kwok, Afshari, García‐Alías, & Fawcett, 2008; Pizzorusso et al., 2002; Romberg et al., 2013).

Levels of the brain‐specific lectican BCAN were significantly elevated in AD patients, contributing to a loss of synaptic plasticity observed prior to neuronal cell death (Howell, Bailey, Cozart, Gannon, & Gottschall, 2015). Aβ protein can directly interact with BCAN in vitro, as well as disrupting the proteolytic cleavage mechanisms involved in BCAN processing, potentially accentuating the inhibition of synaptic plasticity (Ajmo et al., 2010; Howell et al., 2015). Injection of ChABC into the hippocampus of 15‐month‐old double transgenic APPswe/PS1dE9 mice which have greatly increased Aβ protein production and severe synaptic deficits with age, removed the CS chains on lecticans and the effects on Aβ plaques, was monitored. Interestingly, the application of ChABC resulted in a significant reduction in Aβ burden and an increase in synaptic density (Howell et al., 2015). These results introduce the possibility of targeting perisynaptic lecticans as a starting point for an AD therapy. The APPswe/PS1dE9 mice model has also been used to demonstrate the up‐regulation of several ECM proteins including Hapln1, NCAN, BCAN, and Tn‐R that coincides with an early increase in synaptic Aβ levels, as well as LTP and contextual memory impairment. Treatment of the ECM with ChABC was able to reverse these adverse effects, suggesting that increasing ECM levels contributes to early memory deficits in AD (Végh et al., 2014).

The relative sulfation patterns on CSPGs notably influence the formation of PNNs and the effect they have on axon growth. During development and maturation, the ratio of chondroitin‐4‐sulfate (C4S) to chondroitin‐6‐sulfate (C6S) in the PNNs gradually increases (Miyata, Komatsu, Yoshimura, Taya, & Kitagawa, 2012). This is due to both the depletion of C6S over time and the increase in C4S during progression into adulthood (Miyata et al., 2012). The change in C4S/C6S ratio is partly the result of the change in activity of both chondroitin 6‐sulfotransferase‐1 (C6ST1) and chondroitin 4‐sulfotransferase‐1 (C4ST1), Golgi‐resident enzymes which are responsible for the sulfation of unsulfated chondroitin to the 6‐sulfated and 4‐sulfated forms of chondroitin, respectively (Mikami & Kitagawa, 2013; Silbert & Sugumaran, 2002). The changes in sulfation observed during development continue into adulthood and aging where C6S sulfation drops further (Foscarin, Raha‐Chowdhury, Fawcett, & Kwok, 2017). C6S has been shown to be permissive to axonal growth and regeneration (Kitagawa, Tsutsumi, Tone, & Sugahara, 1997; Lin, Rosahl, Whiting, Fawcett, & Kwok, 2011). In contrast, C4S is thought to be the most inhibitory form of CS to axonal growth and guidance (Deepa et al., 2006; Wang et al., 2008). The shift in C4S/C6S ratio is crucial for successful PNN development and restriction of axon growth during the closure of the critical period. If C6S is up‐regulated by overexpression of C6ST1, then PNN formation in the visual cortex is severely impaired and the mice are more plastic (Miyata et al., 2012). On the contrary, reducing C6S level in *c6st1* KO mice shows a reduction in axonal regeneration after a CNS lesion (Lin et al., 2011). An increase in C6S also surprisingly leads to an increase in the proteolysis of ACAN by a disintegrin and metalloproteinase domain with thrombospondin motif (ADAMTS) protease, further disrupting PNN formation (Miyata & Kitagawa, 2016).

Besides using ChABC to digest PNNs, some studies have used hyaluronidase to specifically target HA chains and disrupt the entire PNN (Frischknecht et al., 2009). Happel and colleagues injected hyaluronidase into the auditory cortex of adult Mongolian gerbils and investigated the effects this had on cognitive flexibility in reversal learning. They found that removal of the PNN through this method improved the activity‐dependent reorganisation of existing synaptic networks during reversal learning and an overall increase in synaptic plasticity (Happel et al., 2014). In some cases, ChABC and hyaluronidase have been used in combination to eradicate all traces of the CS and HA in the ECM (Hylin et al., 2013). Enzymic degradation using ChABC and hyaluronidase can be considered a crude tool for modulating the ECM. In the context of treating AD patients, using enzymes such as ChABC is not considered practical for targeting the large volume of an adult brain (Fawcett, 2015). More discrete methods, such as specifically targeting and altering the molecular composition of PNNs, must be used if fine modifications to neuronal plasticity are required (van't Spijker & Kwok, 2017).

Several animal KO and transgenic mouse models have been developed to prevent or reduce the formation of PNNs around neurons. As mentioned previously, removing Hapln1 through gene deletion of *hapln1* has consistently resulted in the attenuation of PNNs (Carulli et al., 2010; Czipri et al., 2003; Romberg et al., 2013). Other link proteins that are found in the PNNs such as the brain link protein 2 (Bral2) have also been removed in KO mouse models to inhibit the development of PNNs (Bekku et al., 2012). A useful alternative approach to PNN degradation was shown through the overexpression of C6S to disrupt the accrual of ACAN via *c6st1* transgenic mice (Miyata & Kitagawa, 2016; Miyata et al., 2012). KO mice lacking the chondroitin sulfate *N*‐acetylgalactosaminyltransferase‐1 (CSGalNAcT‐1) enzyme have been used as an alternative way to interrupt CSPG production. Interestingly, these mice still formed structurally identifiable, albeit abnormal, PNNs (Yoshioka et al., 2017). KOs of the gene encoding the trimeric Tn‐R have been conducted, resulting in the disruption (but not complete removal) of the PNNs. This was thought to be due to the absence of two types of CSPGs (phosphacan and NCAN) in the nets (Haunsoø et al., 2000; Suttkus et al., 2014).

There have been examples of multiple KO mice—notably, work by Geissler et al. (2013) generated quadruple KO mice preventing the expression of tenascin‐C, Tn‐R, BCAN, and NCAN and causing severely shrunken PNNs to form. Recently, a novel animal model was developed in which the levels of ACAN were reduced in vivo through targeted *ACAN* gene deletion. This attenuated the PNNs and was shown to result in reinstating of the juvenile ocular dominance plasticity, as well as providing enhancements in OR memory (Rowlands et al., 2018). Despite the numerous successes seen with transgenic animals in removing PNNs and enhancing plasticity, this method of modulating PNNs has both practical and moral hurdles preventing it from being a viable treatment for neurodegenerative disorders in patients at present.

### 4.2 Blocking PNN action

An alternative route to PNN modulation is targeting the interaction of various diffuse ECM‐affiliated molecules to the core components of the PNNs. For example, as discussed previously, Sema3A has been shown to bind specifically to certain CS moieties on CSPGs. This includes chondroitin‐4,6‐sulfate(C4,6S) but not chondroitin‐2,6‐sulfate (C2,6S), despite both forms being disulfated chondroitins, suggesting that Sema3A binding has sulfation pattern specificity rather than overall sulfation quantity specificity (Corredor et al., 2016; Dick et al., 2013). As a chemorepulsive axon guidance molecule, the presence of Sema3A in the PNNs potentiates PNN inhibition on neurite outgrowth and new synaptic connections (Dick et al., 2013). Low MW compounds can be used to block Sema3A and controllably counteract its inhibitory nature in PNNs on axonal growth. For instance, a selective Sema3A inhibitor, SM‐216289, was identified from a fungal strain fermentation broth and shown to regenerate or preserve injured axons both in vitro and in vivo (Kaneko et al., 2006). Several Sema3A C‐terminus‐derived basic peptides have also been reported to interrupt the Sema3A–CS‐GAG interactions (Corredor et al., 2016). A recent paper by Boggio et al. (2019) used adeno‐associated virus to overexpress the soluble fragment of neuropilin 1, the receptor of Sema3A, resulting in an enhanced ocular dominance plasticity in the visual cortex. Additionally, anti‐Sema3A monoclonal antibodies have been developed (Yamashita et al., 2015); while not being designed to specifically target Sema3A in the PNNs, these antibodies could be probed for their relevance in blocking Sema3A binding to PNNs both in vitro and in vivo.

Another PNN binding protein is orthodenticle homeobox 2 (Otx2). This transcription factor also binds to C4,6S in the PNNs of the PV+ interneurons, is then internalised, and modulates gene expression for the maturation of PV+ neurons. It plays an important role in the commencement and termination of the critical period for plasticity (Bernard & Prochiantz, 2016). Moreover, it is thought that Otx2 can facilitate its own uptake in a positive feedback loop by binding to increasingly thriving PNNs (Beurdeley et al., 2012). The transfer of Otx2 into the PV+ interneurons can be blocked by various peptide and CS mimetics and thus reduce PNNs surrounding the cells. A sequence of amino acids which traverses the N‐terminal domain and homeodomain of Otx2 was described as being a putative GAG binding motif (Beurdeley et al., 2012). This motif contains an RK doublet peptide sequence which has proved to be crucial for CS‐GAG binding. An RK peptide was created, and this peptide was used to outcompete binding of an Otx2 to PNNs in cortical cells and thus prevent the internalisation of Otx2 in vitro and in vivo (Beurdeley et al., 2012). Additionally, the RK doublet interacted specifically with C2,6S and C4,6S, both of which contain carbon 6 sulfation (Beurdeley et al., 2012). This again highlights the importance of sulfation patterns on CSPGs for coordination of the ECM and specifically the PNNs. An alternative approach to blocking Otx2–CS‐GAG interactions has been explored using CS analogues (Despras et al., 2013). The preparation of hexasaccharide C4,6S analogues from lactose was first described and then followed up by in vitro and in vivo studies to assess whether they successfully mimicked natural C4,6S. Using a gel shift assay, C4,6S analogues were shown to bind to Otx2, presumably at the RK doublet peptide sequence (Despras et al., 2013). Additionally, infusion of a particular hexasaccharide C4,6S analogue reduced the Otx2 internalised by PV+ interneurons as well as slightly inhibiting WFA staining, suggesting disruption of the PNNs around these cells (Despras et al., 2013). Lastly, inducing point mutations in the RK doublet motif of the Otx2 gene in knock‐in mice, the localisation and accumulation of Otx2 in the PV+ were disrupted (Lee et al., 2017). Furthermore, this led to a delay in PNN expression and the extension of the critical period for plasticity (Lee et al., 2017).

Recently, antibodies that bind to and block the brush‐like CS chains on PGs in PNNs have been explored in vivo (Figure 3). The Cat316 antibody can specifically recognise C4S, blocking the usual inhibitory effect on axonal growth associated with C4S on CSPGs (Yang et al., 2017). It was also noted that the binding of Cat316 to the PNNs moderately prevented the binding of Sema3A to the PNNs, accentuating the constructive effect on axon growth (Yang et al., 2017). Despite restoring memory function in mice with Tauopathies, this approach did not significantly alter disease progression. Additionally, there remains the necessity to inject Cat316 directly into the brain. In order to address this, the authors suggest that a better blocking agent should be developed that would be able to cross the blood brain barrier, if this approach is to be used in a therapeutic setting in the future for neurodegenerative diseases such as AD (Yang et al., 2017).

There are several known membrane‐bound cell surface CSPG receptors that contribute to the inhibitory nature of CSPGs and control of neural plasticity (Miao, Ye, & Zhang, 2014). These include the receptor protein tyrosine phosphatase σ (PTPσ or RTP Type S) and the leukocyte common antigen‐related phosphatase (LAR or RTP Type F; l; Fisher et al., 2011; Shen et al., 2009), as well as the Nogo receptors, NgR1 and NgR3 (Dickendesher et al., 2012), which have all been reported to have high binding affinity for CSPGs. Axonal growth inhibition from CSPGs was shown to be reduced when a double knockout of the *PTPσ* gene was carried out in neuronal cell culture (Shen et al., 2009). Similarly, dorsal root ganglion (DRG) neurons derived from *LAR* KO mice do not suffer a restriction in neurite outgrowth in the presence of CSPG substrate (Fisher et al., 2011) indicating the importance of the LAR–CSPG interaction in plasticity. To further confirm these results, two sequence‐targeting peptides, extracellular LAR peptide and intracellular LAR peptide, were used to treat CSPG substrate cultured DRG neurons resulting in an increase in neurite length as expected (Fisher et al., 2011). More recently, a membrane‐permeable peptide mimetic was developed and utilised, binding to PTPσ and preventing the interaction with CSPGs (Lang et al., 2015). The peptide mimetic known as intracellular sigma peptide represented the highly conserved wedge domain on PTPσ and treatment of adult sensory neurons with intracellular sigma peptide allowed axons to sprout and cross through a CSPG gradient (Lang et al., 2015). To assess the significance of the Nogo receptors on axon regeneration, several Nogo receptor KO mice were generated and studied (Dickendesher et al., 2012). Following an optic nerve crush injury, the regeneration of retinal ganglion cell axons was assessed in the various mutants. Triple mutant *NgR1* ^*−/−*^ *; NgR2* ^*−/−*^ *; NgR3* ^*−/−*^ mice and double mutant *NgR1* ^*−/−*^ *; NgR3* ^*−/−*^ mice showed improved axon regeneration when compared to wild‐type mice (Dickendesher et al., 2012).

## 5 FUTURE DIRECTIONS

The extensive and thorough research already carried out and presented here has clearly demonstrated the promise in targeting PNNs for the treatment of several neurological conditions. Much of the current methodology for modulating PNNs involves the use of macromolecules such as digestive enzymes or antibodies due to the relative ease of use and production in the early stages of therapy development. Additionally, genetic modifications such as KO models are frequently used to abolish or attenuate PNNs. These methods are very useful to establish the proof of concept that modulating PNNs can have a significant effect on plasticity and memory; however, they are not easy to translate into clinically relevant therapies. Commonly, low MW compounds are developed to try and mimic the effects that macromolecules and/or KO models have on phenotype (Samanen, 2013). There are considerable benefits to using low MW compounds over other forms of therapy such as biologics and genetic modifications. One of the main advantages, especially in the early stages of development, is the ease of optimisation. Making subtle adjustments to biologics to improve the effectiveness is seldom successful due to the size and complexity of these species, whereas modifying low MW compounds can easily be achieved through chemical synthesis; guidance by methods such as structure‐based drug design can aid in improving efficacy of the drug (Samanen, 2013). Additionally, low MW compounds are usually orally administered which is commonly preferred over the parenteral administration required for biologics due to their poorly defined and adverse physiochemical properties (Samanen, 2013).

One of the most obvious targets for the development of low MW compounds would be the transmembrane enzyme HAS. Inhibiting HAS would prevent the synthesis of the HA potentially resulting in the breakdown of PNNs and disrupting the ECM overall. HA synthesis has been successfully targeted before on numerous occasions, albeit in alternative indications to AD (Nagy et al., 2015). The commercially available drug 4‐methylumbelliferone has frequently been used for this purpose, due to its ability to deplete one of the substrates (uridine diphosphate GlcA) required for HA synthesis (Nagy et al., 2015). The fact that HA is found throughout all ECMs and not just in neural ECMs and around the PNNs, coupled with the promiscuity of 4‐methylumbelliferone, means that targeted treatment to specifically disrupt HA synthesis in the PNNs may be a challenge (Garg & Hales, 2004; Nagy et al., 2015).

A promising target for intervention with low MW compounds on PNNs and axonal growth may be the CSPGs. More specifically, preventing the biosynthesis of these structures by targeting the sulfotransferase enzymes that provide the CS chains has been shown to be an effective method to renew axonal growth. In recent studies, the Golgi‐resident *N*‐acetylgalactosamine 4‐sulfate 6‐sulfotransferase (GALNAC4S‐6ST) was shown to be modestly inhibited by a low MW compound that had been optimised using a high throughput screening and medicinal chemistry (Cheung et al., 2017). The most potent compound decreased the overall levels of C4,6S and overall sulfation in vitro as well as reversing the inhibition of axonal growth caused by CSPGs in DRG neurons (Cheung et al., 2017). Despite being selective towards membrane‐bound GAG sulfotransferases compared to cytosolic sulfotransferases, the optimised compound was indiscriminately inhibitory towards several membrane bound GAG sulfotransferases including the closely related C4ST1 (Cheung et al., 2017). This observation suggests that the reversal of inhibited axonal growth seen in DRG neurons could be due to pan‐sulfotransferase inhibition. Clearly, this highlights the potential hurdles involved in achieving drug–protein interaction specificity, which may pose a challenge for the further development of low MW sulfotransferase inhibitors.

There are numerous other components in the PNNs that can potentially be targeted by low MW compounds, including the CNS‐exclusive tenascin, TN‐R, and the HA–CSPG bridging link proteins. Additionally, it can be envisaged that interruption of the binding of the known PNN binding molecules Sema3A and Otx2 may provide success. Further research into the druggability of these proteins is required before an inhibitor can be realised. Other therapeutic avenues may be pursued. However, given the benefits of developing a low MW modulator, as previously mentioned, coupled with the pitfalls frequently encountered with these other modalities, discovery of a low MW drug seems to provide the most promise going forward.

## 6 CONCLUSIONS

Increasingly, the emerging evidence suggests that PNNs have a vital role to play in controlling plasticity, regulating axonal growth and regeneration and memory storage during development and throughout adulthood. This has obvious implications in several neurological diseases, including the many forms of dementia, in which the underlying mechanisms involved in disease progression are not fully understood. The molecular make‐up and function of the PNNs are increasingly being appreciated, when assessing the scientific literature currently available. This understanding has been used to target and disrupt the PNNs using numerous methods to increase neuronal plasticity. This includes enzymic degradation of the nets, genetic therapy to prevent PNN formation, and blocking of PNN action using low MW compounds and/or biomolecules. The abundance of potential protein targets in the PNNs should inspire the development of novel therapeutic agents with a focus on utilising the ease of discovery and optimisation of low MW compounds to inhibit PNNs, in order to reactivate plasticity and restore cognition in neurological disorders such as AD.

### 6.1 Nomenclature of targets and ligands

Key protein targets and ligands in this article are hyperlinked to corresponding entries in http://www.guidetopharmacology.org, the common portal for data from the IUPHAR/BPS Guide to PHARMACOLOGY (Harding et al., 2018), and are permanently archived in the Concise Guide to PHARMACOLOGY 2017/18 (Alexander, Fabbro et al., 2017; Alexander, Kelly et al., 2017; Alexander, Peters et al., 2017).

## CONFLICT OF INTEREST

The authors declare no conflicts of interest.
